# Delineating the activity of the potent nicotinic acetylcholine receptor agonists (+)-anatoxin-a and (−)-hosieine-A

**DOI:** 10.1107/S2053230X22007762

**Published:** 2022-08-09

**Authors:** Holly P. Parker, Alice Dawson, Mathew J. Jones, Rui Yan, Jie Ouyang, Ran Hong, William N. Hunter

**Affiliations:** a Division of Biological Chemistry and Drug Discovery, School of Life Sciences, University of Dundee, Dundee DD1 5EH, United Kingdom; b CAS Key Laboratory of Synthetic Chemistry of Natural Substances, Center for Excellence in Molecular Synthesis, Shanghai Institute of Organic Chemistry, Chinese Academy of Sciences, 345 Lingling Road, Shanghai 200032, People’s Republic of China; Stanford Synchrotron Radiation Lightsource, USA

**Keywords:** acetylcholine-binding proteins, (+)-anatoxin-a, (−)-hosieine-A, crystal structure, ligand-gated ion channels, neurotoxins, nicotine, nicotinic acetylcholine receptors, varenicline

## Abstract

The affinity and thermodynamic parameters for the interactions of two naturally occurring neurotoxins, (+)-anatoxin-a and (−)-hosieine-A, with acetylcholine-binding protein were investigated using a fluorescence-quenching assay and isothermal titration calorimetry. The crystal structures of their complexes with acetylcholine-binding protein from *Aplysia californica* (*Ac*AChBP) were determined and reveal details of molecular recognition in the orthosteric binding site. Comparisons treating *Ac*AChBP as a surrogate for human α4β2 and α7 nicotinic acetylcholine receptors (nAChRs) suggest that the molecular features involved in ligand recognition and affinity for the protein targets are conserved. The ligands exploit interactions with similar residues as the archetypal nAChR agonist nicotine, but with greater affinity. (−)-Hosieine-A in particular has a high affinity for *Ac*AChBP driven by a favorable entropic contribution to binding. The ligand affinities help to rationalize the potent biological activity of these alkaloids. The structural data, together with comparisons with related molecules, suggest that there may be opportunities to extend the hosieine-A scaffold to incorporate new interactions with the complementary side of the orthosteric binding site. Such a strategy may guide the design of new entities to target human α4β2 nAChR that may have therapeutic benefit.

## Abstract 2

The binding of two potent alkaloid neurotoxins to acetylcholine-binding protein was investigated using calorimetry and fluorescence titration. The crystal structures of two complexes and sequence and structure comparisons inform discussion on the biological implications for interactions with human nicotinic acetylcholine receptor subtypes, which are important therapeutic targets.

## 1. Introduction

Natural products have helped to shape modern neuroscience research. Specifically, compounds have been exploited to dissect the structure–function relationships of proteins involved in nervous systems, and to underpin the pharmacological studies that drive modern drug discovery (Prisinzano, 2009 ▸). We were attracted to two natural products, (+)-anatoxin-a and (−)-hosieine-A, which are efficient agonists of nicotinic acetylcholine receptors (nAChRs), and set out to examine the molecular features that allow these relatively small compounds to elicit a potent biological effect.

**Figure 1:**
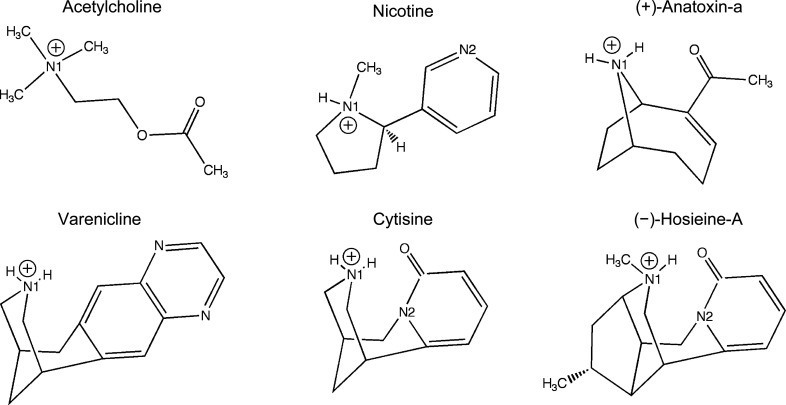
The chemical structures of the physiological ligand of nAChRs (acetylcholine), a potent agonist (nicotine), two drugs that target these ion channels (cytisine and varenicline) and the natural products characterized in this study [(+)-anatoxin-a and (−)-hosieine-A]. The p*K*_a_ of anatoxin-a is 9.4 (Koskinen & Rapoport, 1985 ▸) and under the conditions in which we obtained crystals the ligand is likely to be protonated. The p*K*_a_ values of cytisine, varenicline and nicotine are calculated to be 7.9, 9.7 and 8.5 (http://www.chemspider.com/), respectively, and by inference a similar value should apply to (−)-hosieine-A, hence we show the protonated forms that are expected to predominate at physiological pH.

Anatoxin-a is a semi-rigid bicyclic amine produced by cyanobacteria and is amongst the smallest toxic alkaloids known (Fig. 1 ▸; Carmichael *et al.*, 1975 ▸; Devlin *et al.*, 1977 ▸; Rodgers *et al.*, 2018 ▸). The (+)-enantiomer, a neurotoxin first isolated from freshwater algal blooms, is called ‘very fast death factor’ since it is fatal minutes after intraperitoneal injection in mice (LD_50_ of 200–250 µg kg^−1^; Devlin *et al.*, 1977 ▸; Christensen & Khan, 2020 ▸). The compound is an agonist of muscle and neuronal nAChRs, with the (+)-enantiomer being around 150 times more potent than the (−)-enantiomer (Spivak *et al.*, 1980 ▸; Koskinen & Rapoport, 1985 ▸; Rodgers *et al.*, 2018 ▸). (+)-Anatoxin-a is most active against the α4β2 nAChR subtype, with greater potency than the endogenous neurotransmitter acetylcholine (Thomas *et al.*, 1993 ▸; Molloy *et al.*, 1995 ▸). Radioligand competition binding assays and electrophysiology studies report *K*_i_ values ranging from 1 to 90 n*M* for (+)-anatoxin-a acting on human α4β2 and rat α7 subtypes (Gohlke *et al.*, 2002 ▸; Mazurov *et al.*, 2012 ▸). The (+)-form displays enhanced activity over the (−)-enantiomer when tested against *Torpedo* electric organ in both a radioligand competition assay (IC_50_ values of 85 n*M* and 4.4 µ*M*, respectively) and by patch-clamp electrophysiology (IC_50_ values of 32 n*M* and 1.6 µ*M*, respectively; Swanson *et al.*, 1986 ▸). Successful synthetic routes for racemic as well as chirospecific forms of anatoxin have been developed, although the latter involves a multi-step process with low yields of between 4% and 10% (Koskinen & Rapoport, 1985 ▸; Brenneman & Martin, 2004 ▸).

Similarly, four alkaloids extracted from *Ormosia hosiei*, an ingredient of Chinese herbal medicines, have been characterized (Pouny *et al.*, 2014 ▸). The most abundant of these, (−)-hosieine-A (Fig. 1 ▸), displays high affinity towards neuronal α4β2 nAChRs (*K*_i_ of <1 n*M* in a radioligand competition binding assay) and is more potent than nicotine (Ouyang *et al.*, 2015 ▸). The hosieine scaffold shares a common biosynthetic origin with a number of lupin alkaloids but differs by the incorporation of a 2-azabicyclo[3.2.1]octane system. Recent enantioselective syntheses of (−)-hosieine-A have provided sufficient material for study (Ouyang *et al.*, 2015 ▸; Huang *et al.*, 2018 ▸).

These natural products possess physicochemical properties common to drugs that are able to penetrate the central nervous system. These properties include low molecular mass, few hydrogen-bonding groups and small polar surface areas, limited flexibility and p*K*_a_ values in the range 7.5–10.5 (Pajouhesh & Lenz, 2005 ▸). Furthermore, both compounds share structural features with cytisine (Fig. 1 ▸), a natural product used as a therapeutic smoking-cessation agent and a lead compound in the development of other molecules, such as varenicline, which also fulfill this role (Rouden *et al.*, 2014 ▸; Prochaska & Benowitz, 2016 ▸). These molecules are conformationally restricted, with planar entities, and possess quaternary amine and carbonyl groups in a similar three-dimensional structure. Of note, (−)-hosieine-A, which can be considered to be a cytisine derivative, is rigid, with four of the five asymmetric centers constrained by the bicyclic system.

We sought to investigate the structure–activity relationships of (+)-anatoxin-a and (−)-hosieine-A with their physiological targets, the cation-selective pentameric ligand-gated ion channels (pLGICs) nAChRs. Our findings may support the development of new chemical entities that target nAChR forms, which are highly prized therapeutic targets for a range of disorders (Gotti *et al.*, 2009 ▸; Dineley *et al.*, 2015 ▸; Bertrand & Terry, 2018 ▸). For example, cytisine and varenicline discussed above act on nAChRs. They are generally well tolerated drugs, but unpleasant side effects do compromise their use for some individuals (Prochaska & Benowitz, 2016 ▸; Karnieg & Wang, 2018 ▸). Molecules able to elicit the same influence but at a lower dose could circumvent side-effect issues.

Profound advances in the structural characterization of nAChRs, in particular the α4β2 species, which has the highest affinity for nicotine, have provided significant insight into this receptor family (Gharpure *et al.*, 2020 ▸). However, the experimental difficulties associated with such a system, in terms of characterizing interactions with small ligands, guided us to exploit acetylcholine-binding protein from *Aplysia californica* (*Ac*AChBP) as a surrogate. This highly stable protein, which is found in the cholinergic synapse of gastropods, is an orthologue of the extracellular ligand-binding domain (ECD) of nAChRs (Sixma & Smit, 2003 ▸; Nys *et al.*, 2013 ▸; Sauguet *et al.*, 2015 ▸; Shahsavar *et al.*, 2016 ▸). It is available in recombinant form and has been used, together with the orthologue from *Lymnea stagnalis*, to define aspects of ligand recognition involving the excitatory nAChRs (Celie *et al.*, 2004 ▸; Hansen *et al.*, 2005 ▸; Rucktooa *et al.*, 2012 ▸).

The biological target nAChRs follow the standard structural arrangement of pLGICs, with five subunits creating a central ion pore (Gharpure *et al.*, 2020 ▸). Each subunit possesses an ECD followed by four transmembrane α-helices and intracellular contributions from inter-helical segments. The orthosteric binding site is in the ECD at the interface between two subunits, with one contributing the principal (+) face and one the complementary (−) side (Sixma & Smit, 2003 ▸; Gharpure *et al.*, 2020 ▸). The acetylcholine-binding protein *Ac*AChBP shares 20–25% sequence identity with the ECD of nAChR sequences. In *Ac*AChBP, around 44 residues are involved in the orthosteric binding site and the sequence identities with human nAChRs range from 32% (β2) to 45% (α4 and α7). The sequence and structural similarities allow AChBP to inform on aspects of ligand binding and specificity relating to nAChRs.

We investigated the binding of the natural products to *Ac*AChBP using isothermal titration calorimetry (ITC) and a fluorescence-based assay. Crystallographic analyses produced structures at resolutions of 2.5 and 2.6 Å for the complexes of (+)-anatoxin-a and (−)-hosieine-A with *Ac*AChBP, respectively. Armed with new structural detail, we carried out structural and sequence comparisons to inform on the molecular features that contribute to ligand recognition in the ortho­steric site and to the bioactivity of these natural products acting on human α4β2 and α7 nAChRs.

## 2. Materials and methods

### 2.1. General materials

Chemical reagents were purchased from Sigma–Aldrich and Tocris. The synthesis and characterization of (−)-hosieine-A was reported by Ouyang *et al.* (2015 ▸).

### 2.2. Protein production and purification

A gene encoding *Ac*AChBP with a C-terminal His_6_ tag was cloned into the pFastBac1 vector (ThermoFisher). The amino-acid sequence derived from the *A. californica* genome (https://www.broadinstitute.org/aplysia/aplysia-genome-project) is similar to that of UniProt entry Q8WSF8 except that Val60 and Val155 replace two alanine residues. His-tagged *Ac*AChBP was produced by *Trichoplusia ni* High Five cells (ThermoFisher) grown in Express Five medium until a cell count of 15 × 10^5^ cells ml^−1^ was reached. The culture was infected with 5%(*v*/*v*) virus and left for 48 h. The virus was produced with the Bac-to-Bac system (Invitrogen). Protein purification followed previously published methods, which involved metal-ion immobilized affinity chromatography (Dawson *et al.*, 2019 ▸). Characterization and quality control of the samples involved SDS–PAGE and size-exclusion chromatography. The protein concentration was estimated using a theoretical extinction coefficient (ɛ = 1.471 *M* ^−1^ cm^−1^) calculated in *ProtParam* (Gasteiger *et al.*, 2005 ▸).

### 2.3. Equilibrium fluorescence measurements

Equilibrium fluorescence was monitored at room temperature using a spectrophotometer (LS-55, PerkinElmer) in conjunction with the *FL WinLab* 4.00.03 analysis software. The sample (2 ml *Ac*AChBP at 10 µg ml^−1^ in 50 m*M* Tris–HCl pH 7.5, 250 m*M* NaCl; buffer *A*) was excited at 280 nm and the emission intensity was monitored between 300 and 400 nm. The excitation and emission slit widths were set to 5 nm and a detector voltage of 750 V was used. Titrations of ligand into buffer (also used to check the intrinsic ligand fluorescence), of buffer into buffer and of buffer into protein were carried out to provide appropriate controls. Ligands were titrated at 1–2 µl per measurement. Nicotine is highly soluble and was used in the range 1–10 µ*M* in buffer *A*. Stock solutions of anatoxin-a and hosieine-A were prepared in DMSO; these were diluted in buffer *A* and then tested in the ranges 0.1–5 µ*M* and 0.05–1 µ*M*, respectively. The concentration of DMSO during the titrations did not exceed 0.3%. Emission measurements at 337 nm were used to estimate *K*_d_ values from plots of change in percentage fluorescence versus ligand concentration. Data analyses were carried out using *GraphPad Prism*. The data derived from the association of (−)-hosieine-A were analyzed using the specific binding with the Hill slope option and the data derived from association with (+)-anatoxin-a were analyzed using a one-site, specific binding model.

### 2.4. Isothermal titration calorimetry

Titrations were carried out with a MicroCal PEAQ-ITC (Malvern Instruments) at 25°C (298 K). Protein samples (at a concentration of 25 µ*M*) were titrated with ligands using a concentration range of 150–500 µ*M*. Protein samples were prepared such that the buffer and the amount of DMSO present matched those in the titrant. Ligands were prepared from a 100 m*M* stock in DMSO and were diluted to the desired concentration with buffer *A*. An initial injection of 0.4 µl was made to remove any air bubbles in the syringe, followed by injections of 2.0 µl for 17 and 18 injection protocols, respectively. Injections of titrant occurred at 150–180 s intervals with stirring at 750 rev min^−1^. Raw data peaks were integrated and fitted using the nonlinear least-squares method with a single binding-site model. For anatoxin-a, the concentration was adjusted to reflect a 1:1 stoichiometry of enantiomers. Control experiments were established in which the ligand was titrated into buffer, buffer into buffer and buffer/DMSO into protein solution. Titrations were carried out in triplicate and data analyses used the software provided with the instrument.

### 2.5. Crystal growth, data collection and processing

Protein samples at a concentration of 4 mg ml^−1^ were incubated with the ligands (2 m*M*) at room temperature for 1 h before being mixed at a 1:2 ratio of protein:reservoir solution. Well ordered, multi-faced prisms of the *Ac*AChBP–ligand complexes were then grown over a period of 3–4 days at 18°C (291 K) using the hanging-drop method (Table 1 ▸). For the *Ac*AChBP–(+)-anatoxin-a complex the reservoir consisted of 8% PEG 4000 (80 µl of a 50% stock), 0.1 *M* sodium acetate (53 µl of a 1 *M* stock) pH 4.6 and 367 µl ultrapure water. Crystals with a maximum dimension of 25 µm were obtained. The reservoir condition for the *Ac*AChBP–(−)-hosieine-A complex was 0.2 *M* calcium chloride (100 µl of a 1 *M* stock), 0.1 *M* sodium acetate pH 4.55 (50 µl of a 1 *M* stock), 16% 2-propanol [160 µl of a 50%(*v*/*v*) stock] and 190 µl ultrapure water. These crystals attained a maximum dimension of 20 µm.

The crystals were cooled in a stream of nitrogen and an in-house X-ray source (a Rigaku MicroMax-007 HF X-ray generator equipped with VariMax Cu-VHF optics), a Saturn 944HG+ CCD detector and an AFC-11 four-axis partial χ goniometer were utilized for data collection. The diffraction data were integrated using *DIALS* (Winter *et al.*, 2018 ▸) incorporated into *xia*2 (Winter, 2010 ▸) and *XDS* (Kabsch, 2010 ▸) and were scaled and merged using *AIMLESS* (Evans & Murshudov, 2013 ▸). Statistics are presented in Table 2 ▸.

### 2.6. Structure determination and refinement

Structures were solved by molecular replacement using *Phaser* (McCoy *et al.*, 2007 ▸). The initial polypeptide model for the *Ac*AChBP–(+)-anatoxin-a complex structure comprised two pentamers of an *Ac*AChBP structure (PDB entry 2byn; Davis *et al.*, 2020 ▸). Once refined, the polypeptide of the complex provided the starting model for the isomorphous *Ac*AChBP–(−)-hosieine-A structure. The monoclinic crystal form contains two pentameric assemblies in the asymmetric unit. Real-space refinement and model adjustments were carried out using *Coot* (Emsley *et al.*, 2010 ▸) in conjunction with rounds of reciprocal-space refinement using *REFMAC*5 (Murshudov *et al.*, 2011 ▸)*.*

Noncrystallographic symmetry (NCS) restraints were maintained during refinement of both of the reported structures. This decision was based on two factors; firstly, inspection of all NCS-related subunits with electron-density and difference density maps using the ‘NCS ghost’ option in *Coot* and, secondly, divergence of the *R* factors if NCS restraints were released. Although the employment of NCS restraints during refinement is a self-fulfilling prophecy and imposes similarity in each subunit, we are confident that this model is representative of the electron-density and difference density maps and therefore that it is justified to detail only a single orthosteric binding site.

Positive and negative features in difference density and electron-density maps around the Cys207–Cys208 disulfide suggest that partial bond breakage has occurred. In subunit *A* of the *Ac*AChBP–(+)-anatoxin-a complex this was modeled using two rotamers that represent oxidized and reduced forms of the disulfide. No reducing agents were present during the protein purification or crystallization, and therefore the disulfides may be showing signs of radiation damage that might be explained by the collection of highly redundant data. *RADDOSE* (Bury *et al.*, 2018 ▸) was used to estimate the radiation dose that the samples were exposed to during data collection. Assuming a flux of 5 × 10^9^ photons s^−1^, based on the manufacturer’s specifications, the values are 1.9 MGy for the *Ac*AChBP–(+)-anatoxin-a complex and 0.38 MGy for the *Ac*AChBP–(−)-hosieine-A structure.

Many of the programs used are part of the *CCP*4 crystallographic software suite (Winn *et al.*, 2011 ▸). The model quality and the progress of the refinements were monitored using *MolProbity* (Chen *et al.*, 2010 ▸) and the validation tools in *Coot*. Ligand restraint dictionaries were generated using the *Grade* server (Smart *et al.*, 2014 ▸) and these compounds were modeled into difference electron-density maps after several rounds of solvent identification and refinement had been carried out. *Coot* was used to add H atoms to (−)-hosieine-A. The structures and diffraction data have been deposited in the PDB with accession codes 6sh0 and 6sgv for the (+)-anatoxin-a and (−)-hosieine-A complexes, respectively; selected statistics are presented in Table 3 ▸.

### 2.7. Sequence and structure comparisons

Multiple sequence alignments were carried out with *Clustal Omega* (https://www.ebi.ac.uk/Tools/msa/clustalo/). FASTA sequences were obtained from UniProt (see above). The structure of the human α4_2_β2_3_ nAChR (PDB entry 6cnj; Walsh *et al.*, 2018 ▸) was used for structural alignments in *Coot* using the secondary-structure matching option.

## 3. Results and discussion

We use the numbering of the full-length amino-acid sequences of *Ac*AChBP (UniProt code Q8WSF8; http://www.uniprot.org/) and human nAChR α4 (UniProt code P43681), α7 (UniProt code P36544) and β2 (UniProt code P17787) subtypes.

A structure of a highly modified *Ac*AChBP–(+)-anatoxin-a complex is present in the Protein Data Bank (PDB entry 4zjs; Luo *et al.*, 2009 ▸) but no binding data are presented.

### 3.1. Binding studies

Fluorescence measurements and ITC, with nicotine being used as a control and for comparison, provided data on the binding parameters (Table 4 ▸, Supplementary Figs. S1 and S2). The reported *K*_d_ for nicotine binding to *Ac*AChBP is 0.250 µ*M* using a radioligand displacement assay and 0.245 µ*M* using a fluorescence assay (Hansen *et al.*, 2005 ▸). We recorded *K*_d_ = 0.41 ± 0.01 µ*M* in our tryptophan fluorescence assay. With ITC a higher *K*_d_ of 2.30 ± 0.19 µ*M* was observed, with the molar ratio being close to 0.4:1. In direct comparison, others have reported that ITC gave a *K*_d_ of 0.84 µ*M* for nicotine binding to *Ac*AChBP with a similar molar ratio (Rucktooa *et al.*, 2012 ▸). With anatoxin-a, *K*_d_ values of 0.30 ± 0.03 µ*M* (molar ratio of 0.5:1) and 0.15 ± 0.01 µ*M* were obtained with ITC and fluorescence measurements, respectively. An enantiomeric mixture of anatoxin-a was used in these experiments. The properties of two components of such mixtures can be resolved by ITC under optimal conditions, where the form of the titration curve can be dependent on the difference in the affinity of each enantiomer for the target (Fokkens & Klebe, 2006 ▸). However, there is a strict requirement for the difference in affinity to fall within a narrow range. Although a racemic mixture was used, we did not observe an obvious two-step curve and interpreted the data to indicate preferential binding of one enantiomer. It is likely, based on the data described above, that the (+)-form displays a higher affinity for *Ac*AChBP and the binding parameters relate to that molecule. For (−)-hosieine-A the *K*_d_ values are 0.025 ± 0.005 µ*M* (molar ratio 0.7:1) and 0.040 ± 0.001 µ*M*. These values indicate a much higher affinity for the protein compared with (−)-cytisine, for which ITC-derived *K*_d_ values of 1.6 µ*M* and 0.60 ± 0.03 µ*M* are reported (Table 4 ▸; Rucktooa *et al.*, 2012 ▸; Davis *et al.*, 2020 ▸). For comparison, varenicline is reported to have a *K*_d_ of 0.34 µ*M* obtained by ITC (Rucktooa *et al.*, 2012 ▸).

It has previously been noted based on ITC data (Celie *et al.*, 2004 ▸; Jones *et al.*, 2020 ▸) that some ligands, for example carbamylcholine, bind *Ac*AChBP with *N* values of around 0.5. Similar observations are made in the present work, with *N* values of 0.4–0.7 being noted (Table 4 ▸). The crystal structures described below show full occupancy of the ten orthosteric binding sites in the asymmetric units and provide no evidence for cooperativity or allosteric transitions in *Ac*AChBP, so the reason for the low molar ratio for some ligands remains unclear. The ITC data (Table 4 ▸) reveal that the binding of the four ligands to *Ac*AChBP is an exothermic event that is dominated by a favorable enthalpic contribution. The entropic contributions to binding for nicotine, (−)-cytisine, (+)-anatoxin-a and varenicline (Rucktooa *et al.*, 2012 ▸) are un­favorable, but those for (−)-hosieine-A are favorable. Binding of (−)-hosieine-A shows the lowest contribution to binding from enthalpic terms, but it is the favorable entropic term that promotes a greater affinity for the target than those displayed by the other compounds. This is a distinctive property of (−)-hosieine-A compared with the other ligands in the present work. Such an observation is, however, not unique to (−)-hosieine-A. We previously noted that the binding of another natural product, strychnine, to *Ac*AChBP involves a favorable entropic contribution comparable to that observed for (−)-hosieine-A (−1.9 ± 0.5 kcal mol^−1^; Dawson *et al.*, 2019 ▸). That these two ligands share structural features and display similar thermodynamic properties in their interaction with *Ac*AChBP suggested that together they might offer clues to the key features of the interaction with the protein.

Two main factors determine the entropy terms associated with protein–ligand interactions (Singh & Warshel, 2010 ▸ and references therein). These are the changes in conformational and solvation-associated entropy. The changes in conformational entropy are often unfavorable due to the loss of degrees of freedom for either the protein or the ligand or both. Since (−)-hosieine-A is a highly constrained structure, as indeed is strychine (Dawson *et al.*, 2019 ▸), and, as we will detail below, these ligands bind deep in the orthosteric site, interacting with relatively well ordered parts of the protein structure, then we judge that changes in conformational entropy are likely to make only a limited unfavorable contribution to ligand binding. The change in the solvation-associated entropy involves hydrophobic and polar effects as the water structure within a protein binding cavity is displaced by the ligand and as the waters associated with a ligand in solution are displaced during binding to the protein. We judge it likely that the desolvation of (−)-hosieine-A and strychnine and of the *Ac*AChBP binding site may dominate the entropic contribution to the binding event for these two ligands.

The data derived from fluorescence measurements on the titration of *Ac*ChBP with (−)-hosieine-A (Supplementary Fig. S1) were fitted using the specific binding with Hill slope option. The Hill coefficient was determined to be 2.1 (±0.07). Whilst such a value suggests a degree of positive cooperativity (Weiss, 1997 ▸), the structure of the (−)-hosieine-A complex shows only a single well defined ligand occupying the orthosteric binding site and, as described in Section 2.6, we do not observe any differences in the ten orthosteric binding sites in the asymmetric unit that might indicate any structural basis for cooperativity.

Allosteric control contributes to pLGIC function and has been studied extensively in nAChRs (Taly *et al.*, 2014 ▸; Chatzidaki & Millar, 2015 ▸; Delbart *et al.*, 2017 ▸). The role of Ca^2+^ is of note. This cation increases the affinity for agonists and potentiates their activity on nAChRs, producing an increase in current amplitudes. Multiple sites on nAChR structures are implicated in Ca^2+^ binding, which is indicative of complex regulation (Galzi *et al.*, 1996 ▸). Although we derived the structure of the (−)-hosieine-A complex using crystals grown in the presence of Ca^2+^ (Table 1 ▸), we did not identify any positions for these divalent cations. Our structures do not add anything new to the understanding of allosteric regulation in nAChRs.

### 3.2. Crystallographic analyses

**Figure 2:**
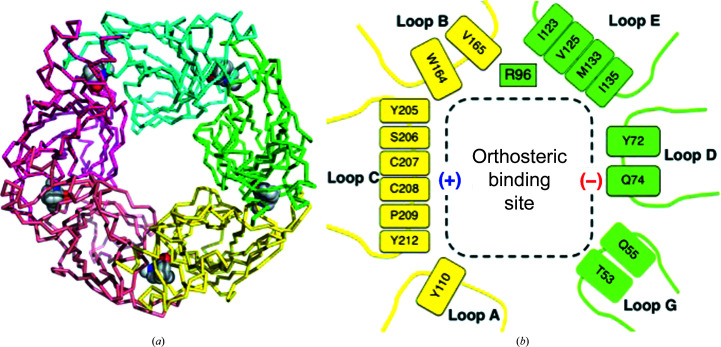
(*a*) A C^α^ trace of the *Ac*AChBP pentamer. Different colors distinguish the subunits. The positions of the orthosteric sites are indicated by (−)-hosieine-A depicted as van der Waals spheres with C atoms in gray, O atoms in red and N atoms in blue. (*b*) A schematic showing the loops and key residues of the orthosteric site formed between the yellow (+) and green (−) subunits, which contribute the principal and complementary sides, respectively.

Isomorphous monoclinic crystals were obtained for the complexes, with an asymmetric unit consisting of two pentameric assemblies, and the relevant statistics are presented in Table 3 ▸. In *Ac*AChBP, as in pLGICs, the orthosteric binding site is constructed at the subunit–subunit interface by seven loops or polypeptide segments (Fig. 2 ▸). Three loops (labeled A–C) form the (+) or principal side and four (D–G) form the (−) or complementary side (Fig. 2 ▸ *b*). In *Ac*AChBP the loops create a narrow hydrophobic cavity dominated by five aromatic residues on one side, a disulfide bond and four aliphatic residues on the other. Residues on loop F are distant from the bound ligands and are omitted for the purpose of clarity. Arg96, although not on any of the assigned seven loops, is included in our analysis since the length of the side chain places a polar group to contribute to the organization of the binding site.

Strong electron density is present in each of the ten binding sites in the asymmetric unit and the ligands refined with average *B* factors that were lower than the values noted for the associated subunits (Supplementary Table S1). A high degree of NCS was evident and thus was maintained in the refinement calculations (discussed in Section 2.6). The orientation of each ligand and the pattern of interactions are internally consistent, so we only detail one orthosteric site for each complex. The observation of some radiation damage to the Cys207–Cys208 disulfide is not considered to be significant.

An enantiomeric mixture of anatoxin-a was used for crystallization, and both forms were tested in modeling into the electron density at a late stage of refinement. The fits to difference density maps were insufficient to distinguish (+) or (−) enantiomers or indeed if a mixture was present. However, the chemical interactions with the (+)-form are plausible, whilst the (−)-form did not match the chemical properties of the binding site well. With the (+)-form well aligned hydrogen bonds are formed from conserved waters and Trp164 carbonyl groups to the ligand carbonyl (2.81 ± 0.12 Å) and onium groups (3.06 ± 0.10 Å). For the (−)-form the distances between these donor and acceptor groups lie between 4.2 and 4.9 Å. This observation is consistent with previous binding/activity studies described above and since our ITC data did not suggest a comparable affinity of the enantiomers, the complex structure incorporated only the (+)-form of the alkaloid.

We also considered data on the conformation of the ligand itself. Solution NMR and force-field calculations suggest that *cis*- and *trans*-chair conformations of this alkaloid are similar in energetic terms, with an approximate ratio of 3:1, and the acetyl side chain is relatively free to rotate (Thompson *et al.*, 1992 ▸). Single-crystal X-ray diffraction analyses of an acetyl derivative (Huber, 1972 ▸) and (+)-anatoxin-a itself (Koskinen & Rapoport, 1985 ▸) show the *trans*-chair conformation. In our model the chair conformation fits the density well and the plausible chemical environments for methyl and carbonyl groups are consistent with a *trans* configuration of (+)-anatoxin-a (Figs. 1 ▸ and 3 ▸).

The (+)-anatoxin-a molecule binds deep in a hydrophobic environment, interacting primarily with residues on the principal side of the binding site. Here, the orthosteric site is dominated by the presence of aromatic residues (Figs. 2 ▸ and 3 ▸). The protonated amine donates a hydrogen bond to the Trp164 carbonyl and is positioned to participate in a cation–π interaction with the indole, a common and important feature of nAChR complexes (Zhong *et al.*, 1998 ▸). The side chain of Trp164 is held in position by a hydrogen bond to the carbonyl of Ile135. Four tyrosine residues (Tyr72, Tyr110, Tyr205 and Tyr212) participate in van der Waals interactions with the azobicyclo component of the ligand. Tyr72 may contribute to cation–π interactions. In some complexes between *Ac*AChBP and tertiary amines, for example epibatidine derivatives (Bueno *et al.*, 2022 ▸), the hydroxyl group of Tyr110 forms a hydrogen bond to the amine. In the (+)-anatoxin-a complex the separation (over 4 Å) and orientation of the functional groups precludes such an interaction. Rather, the Tyr110 side chain appears to be fixed by a hydrogen bond to the carbonyl of Ser163 and an ordered water, which in turn forms hydrogen bonds to the carbonyl of Ile213 and then participates in a network of ordered water molecules forming bridges through to Tyr205 and Asp214 (not shown). There are van der Waals interactions between part of the bicyclic ring system and the neurotoxin methyl group and Cys207 and Cys208, and also between one side of the ethane-1-one and Val125, Met133 and Ile135 and between the other side and Val165. The (+)-anatoxin-a carbonyl accepts a hydrogen bond from a water molecule that in turn participates in hydrogen bonds to the main-chain amide of Ile135 and the carbonyl of Ile123. This hydration feature is common to other structures, for example AChBP–nicotine complexes (Sauguet *et al.*, 2015 ▸; Shahsavar *et al.*, 2016 ▸; Bueno *et al.*, 2022 ▸). The (+)-anatoxin-a O atom occupies almost exactly the same position as the pyridine N atom of the agonist nicotine, representing an overlap of two hydrogen-bond acceptors in the orthosteric site.

(−)-Hosieine-A occupies the same position as (+)-anatoxin-a, interacting with the same components of *Ac*AChBP. An overlay reveals that the protein structures of the two complexes are similar, with the exception of conformational changes to the side chains of Tyr72, Tyr110 and Tyr212 and a slight outward movement of loop C. These tyrosine side chains adjust their position due to the influence of the (−)-hosieine-A methyl substituents. In the presence of this natural product the side chain of Tyr72 adopts a different rotamer and the hydroxyl group is displaced 4.2 Å towards loop D due to the position of the methyl substituent on the five-membered ring. New hydrogen bonds are formed between Tyr72 OH and Gln55 and the main-chain carbonyl of Tyr110, as the interaction with Ser184 OG is lost. Due to the position of the methyl substituent on the quaternary amide, the hydroxyl group of Tyr110 is pushed towards loop B by around 2.5 Å, replacing a water molecule observed in the (+)-anatoxin-a complex. There is now potential for a hydrogen bond between Tyr110 and the main-chain carbonyl of Ile213. Although the quaternary groups of the two toxins are involved in a hydrogen bond with the main-chain carbonyl of Trp164 and cation–π interactions with the side chain, they are approximately 2.2 Å distant in an overlay as distinct features of the ligands have to be accommodated in the binding site. At the other end of the natural products, the carbonyl groups are within 0.9 Å of each other, forming similar interactions, in particular the hydrogen bond to the ordered and highly conserved water molecule that also links to the carbonyl of Ile123 and the amide of Ile135. As noted for the (+)-anatoxin-a complex, this ligand O atom maps almost exactly to the pyridine N atom of nicotine when this agonist is bound to *Ac*AChBP. Tyr212 is positioned by accepting a hydrogen bond from the side chain of Arg96, a feature that may help to order the binding site and remains preserved when (+)-anatoxin-a is bound. In the (−)-hosieine-A complex, a small adjustment, by about 0.6 Å, of Tyr212 is noted.

**Figure 3:**
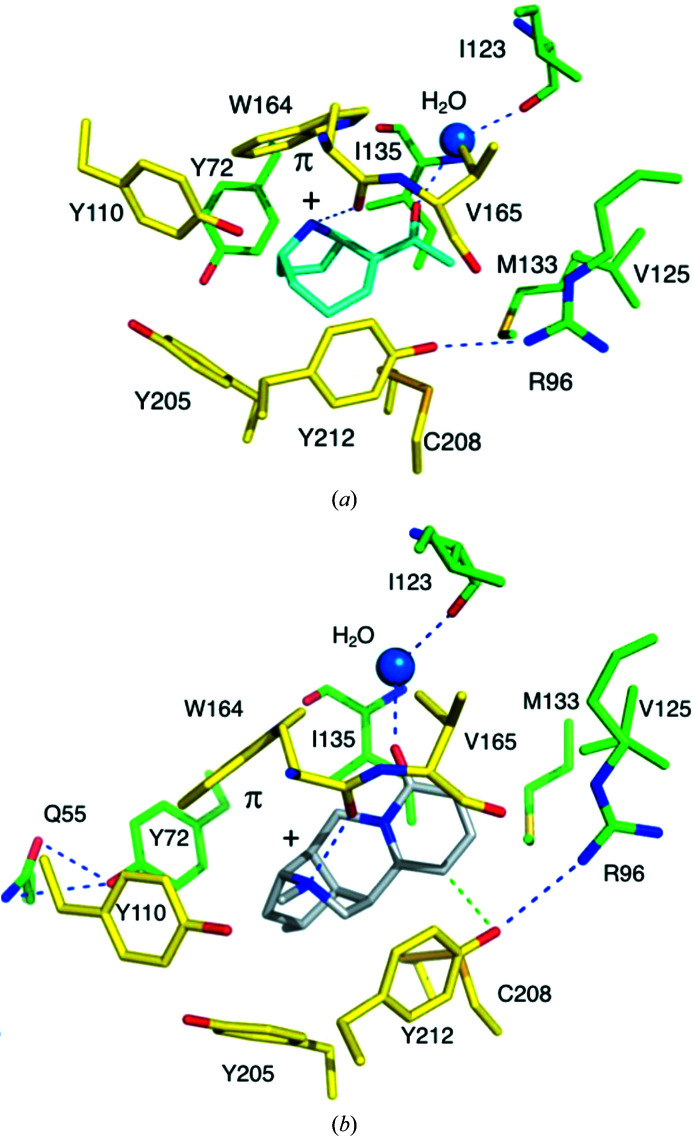
Ligands and key residues in the orthosteric site of *Ac*AChBP. The positions of O atoms are red, of N atoms are blue and of S atoms are dark yellow; those of C atoms of residues on the principal side are yellow and those on the complementary side are green, as in Fig. 2 ▸. Cys207, the disulfide partner of Cys208, is not labeled. A water molecule is shown as a marine-colored sphere. Blue dashed lines are potential hydrogen bonds. (*a*) (+)-Anatoxin-a with C atoms in cyan. (*b*) (−)-Hosieine-A with C atoms in gray. A potential C—H⋯O hydrogen bond between the natural product and Tyr212 is shown as a green dashed line.

The position of the Tyr212 hydroxyl suggests the presence of a C—H⋯O hydrogen bond to (−)-hosieine-A (Fig. 3 ▸ *b*). Surveys of accurate small-molecule neutron diffraction crystal structures (Taylor & Kennard, 1982 ▸) and of protein–ligand complexes in the Protein Data Bank (Itoh *et al.*, 2019 ▸) provide a basis for assigning this interaction as a C—H⋯O hydrogen bond. In the ten orthosteric binding sites of the *Ac*AChBP–(−)-hosieine-A complex the mean C–O distance is 3.12 (±0.06) Å and the mean O–H distance is 2.42 (±0.08) Å. The mean C—H—O angle is 128.9 (±6.9)°. These values are well within the ranges reported (Itoh *et al.*, 2019 ▸).

There are two crystal structures with cytisine in the PDB that are relevant to our study. The *Ac*AChBP–cytisine structure (PDB entry 4bqt; Rucktooa *et al.*, 2012 ▸) is of low resolution (2.9 Å) and does not show water molecules in the binding site. However, the higher resolution engineered *Ac*AChBP–cytisine structure (PDB entry 5syo; J. Bobango, J. Wu, I. T. Talley & T. T. Talley, unpublished work) with loop C altered to mimic that of the human α_3_ nAChR structure shows a water molecule in the same position hydrogen-bonded to the cytisine carbonyl and the complementary side residues.

The solvent-accessible surface areas of nicotine, (+)-anatoxin-a and (−)-hosieine-A, when considered in isolation, are 333, 317 and 390 Å^2^, respectively. When bound to *Ac*AChBP these ligands lose 99.9%, 98.8% and 96.6% of this surface, respectively. In essence, these natural products are buried deep in the orthosteric site, occluded from bulk solvent, with affinity for *Ac*AChBP. The high affinity that (−)-hosieine-A displays for *Ac*AChBP is likely due in part to the alkaloid being a rigid entity with complementarity of shape to areas of the orthosteric site that are themselves less mobile, hence there is less of a penalty to the free energy of binding due to any change in conformational entropy. This is assisted by the positioning of a few polar groups on the ligand with features on the protein that assist the alignment of the molecule in the binding site. These features serve to promote the van der Waals interactions that result between the ligand methyl and methylene groups with Tyr72, Tyr110 and Tyr205 in loop C.

### 3.3. Extending from *Ac*AChBP to human nAChR binding sites to guide molecular editing

**Figure 4:**
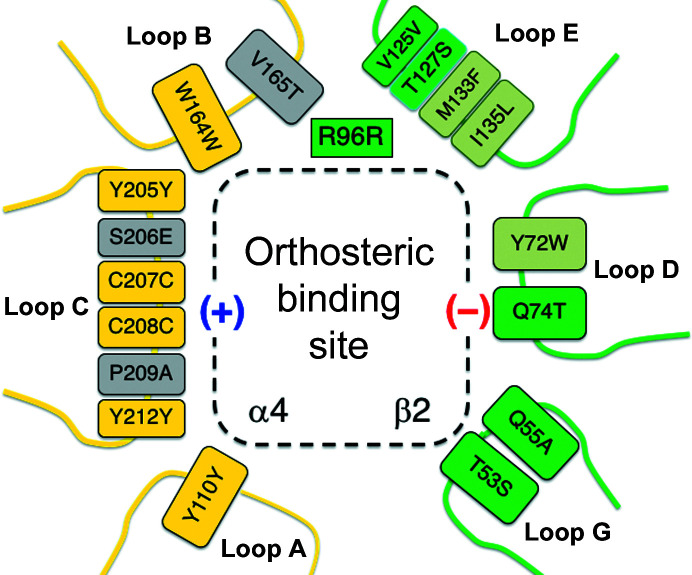
A schematic of the orthosteric binding site comparing key residues of *Ac*AChBP and corresponding amino acids in the nAChR α4(+)β2(−) heteromeric site. Loop F is out of the range of the ligands discussed in this work and has been omitted. Arg96 is included (see text). On the α4(+) side boxes for selected amino-acid positions are colored yellow to highlight strict conservation and gray where the sequence changes. On the β2(−) side the boxes are colored green. Three key positions implicated in the ion-channel response to ligand binding and that are discussed in the text are shown in light green.

Functional nAChRs can be homopentameric (α7) or heteropentameric (for example α4/β2), where an α subunit most commonly forms the principal side, with an α or β subunit providing the complementary side. The α4/β2 heteropentamer contains two α4(+)/β2(−) binding sites, which are key to function. Comparisons of the *Ac*AChBP complexes with cryo-EM structures of nAChR in complex with nicotine (PDB entry 6cnj; Walsh *et al.*, 2018 ▸), with varenicline (PDB entry 6usf; Mukherjee *et al.*, 2020 ▸) and with sequences that correspond to the orthosteric site of human heteromeric nAChR α4(+)/β2(−) were carried out. The conservation of sequence and structure, in particular the aromatic cage of the binding site that is so important for ligand binding, suggests that the orientations of the natural products and the interactions that they form with *Ac*AChBP are representative of what occurs with nAChRs. In the *Ac*AChBP complexes we identified 13 residues that are key to interaction with the natural products (Fig. 3 ▸). Of these, eight are strictly conserved in the α4(+)/β2(−) site (Arg96, Tyr110, Val125, Trp164, Tyr205, Cys207, Cys208 and Tyr212). A sequence alignment of the orthosteric binding-site loops of *Ac*AChBP and the human α4, α7 and β2 nAChR forms is presented in Supplementary Fig. S3. The differences from the human receptor type are Tyr72Trp, Ile123Gln, Met133Phe, Ile135Leu and Val165Thr substitutions. These are conservative changes, except for Ile123Gln (Fig. 4 ▸). However, in this case it is the main-chain carbonyl accepting a hydrogen bond from a highly ordered water molecule that links to the ligands, therefore the identity of the side chain is less important. The differences between *Ac*AChBP and a homomeric α7(+)/α7(−) orthosteric site are similar to those just described, with five of the 13 key residues changing. The residues that differ are Tyr72Trp, Ile123Gln, Met133Gln, Ile135Leu and Val165Ser. The Met133Gln difference, on the side of the binding site, is unlikely to have a major influence given that the bulk of the side chains are similar, and the methionine side chain, like that of glutamine, can participate in hydrogen-bonding inter­actions.

It would be desirable to have compounds that act as nAChR subtype-specific partial agonists. Such compounds, which are able to bind nAChR α4(+)β2(−) with high affinity to desensitize and induce the ion channel to open, offer potential therapeutic benefit for a range of disorders and for the control of pain (Gotti *et al.*, 2009 ▸; Dineley *et al.*, 2015 ▸; Bertrand & Terry, 2018 ▸). Varenicline is already in clinical use and structure–sequence comparisons are informative. Varenicline has been characterized in complex with *Ac*AChBP (Rucktooa *et al.*, 2012 ▸) and with the homologue from *Capitella teleta* (Billen *et al.*, 2012 ▸). Direct comparison with the (−)-hosieine-A structure that we report indicates that the compounds interact with the proteins in much the same way; indeed, an overlay positions the (−)-hosieine-A O atom about 0.8 Å from a varenicline N atom; these are two hydrogen-bond acceptor groups that participate in the same interactions. The ligands place the charged group in the aromatic cage, with the rest of the compound directed towards the complementary (−) side of the orthosteric site. These structures draw attention to residues on loops D and E. Three residues on these two loops are important determinants of ligand effects on the nAChR α4(+)β2(−) ion channels (Billen *et al.*, 2012 ▸). The amino-acid positions that have been highlighted are Tyr72Trp on loop D and Met133Phe and Ile135Leu on loop E. A future avenue of research might be to edit the (−)-hosieine-A framework to engage in specific interactions with loops D and E of the nAChR β2(−) structure. In this way the high affinity of (−)-hosieine-A, driven by the strong entropic contribution to binding arising through interaction with the aromatic cage, provides a good starting point for generating high-affinity ligands.

## 4. Conclusions

We sought to understand the chemical features and inter­actions that allow two alkaloid neurotoxins to influence nAChR ion channels. Orthogonal binding assays, with calorimetric and fluorescence measurements, were applied using *Ac*AChBP, a structural homolog. To complement the binding data, X-ray crystallographic methods were applied to determine structures, and these were compared with complexes of other ligands with *Ac*AChBP [for example nicotine, (−)-cytisine and varenicline]. The data are compared with literature values, sequence and structural data on the heteromeric human α4β2 nAChR (Walsh *et al.*, 2018 ▸) to provide an understanding of the molecular features that control the biological efficacy of these two potent natural products.

The development of varenicline from the lead compound (−)-cytisine involved a modification to the ligand that influences its interactions with the (−) face, which is the complementary side of the orthosteric site. Our structural and binding data suggest that in a similar fashion there may be opportunities to develop partial agonists with high binding affinities based on the hosieine-A chemical scaffold with relatively small alterations that target the complementary side of the binding site.

## Supplementary Material

PDB reference: *Ac*AChBP, complex with anatoxin-a, 6sh0

PDB reference: complex with (−)-hosieine-A, 6sgv

Supplementary Figures. DOI: 10.1107/S2053230X22007762/dp5128sup1.pdf

## Figures and Tables

**Table 1:** Crystallization conditions of the two complexes analyzed

Complex	(+)-Anatoxin-a	(−)-Hosieine-A
Method	Vapor diffusion	Vapor diffusion
Plate type	Hanging drop (Hampton Research)	Hanging drop (Hampton Research)
Temperature (K)	298	298
Protein concentration (mg ml^−1^)	4	4
Buffer composition of protein solution	50 m*M* Tris–HCl, 250 m*M* NaCl pH 7.5	50 m*M* Tris–HCl, 250 m*M* NaCl pH 7.5
Composition of reservoir solution	8% PEG 4K, 0.1 *M* sodium acetate pH 4.6	0.2 *M* CaCl_2_, 0.1 *M* sodium acetate pH 4.5, 16% 2-propanol
Volume and ratio of drop	1 µl protein + 2 µl reservoir solution	1 µl protein + 2 µl reservoir solution
Volume of reservoir (µl)	500	500

**Table 2:** Data-collection and processing statistics for the two crystal structures presented Values in parentheses are for the highest resolution shell.

Complex	(+)-Anatoxin-a	(−)-Hosieine-A
Diffraction source	Rigaku MicroMax-007 HF rotating anode	Rigaku MicroMax-007 HF rotating anode
Wavelength (Å)	1.5418	1.5418
Temperature (K)	100	100
Detector	Rigaku Saturn 944HG+ CCD	Rigaku Saturn 944HG+ CCD
Crystal-to-detector distance (mm)	60	60
Rotation range per image (°)	0.5	0.5
Total rotation range (°)	1548	295
Exposure time per image (s)	30	30
Space group	*C*2	*C*2
*a*, *b*, *c* (Å)	211.97, 129.87, 131.32	209.80, 133.41, 131.16
α, β, γ (°)	90, 103.17, 90	90, 102.51, 90
Resolution range (Å)	127.87–2.50 (2.54–2.50)	45.03–2.60 (2.64–2.60)
Total No. of reflections	1400309 (53947)	677075 (32998)
No. of unique reflections	119138 (5811)	108313 (5354)
Completeness (%)	99.5 (99.3)	100.0 (99.9)
Multiplicity	11.8 (9.3)	6.3 (6.2)
〈*I*/σ(*I*)〉	7.4 (1.8)	6.0 (2.2)
CC_1/2_	0.982 (0.813)	0.986 (0.776)
*R*_merge_ †	0.238 (0.701)	0.261 (1.048)
*R*_p.i.m._ ‡	0.089 (0.286)	0.104 (0.418)
Overall *B* factor from Wilson plot (Å^2^)	23.0	14.5

† *R*_merge_ = 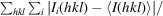 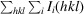 , where *I_i_* (*hkl*) is the intensity of the *i*th measurement of reflection *hkl* and 〈*I*(*hkl*)〉 is the mean value of *I_i_* (*hkl*) for all *i* measurements.

‡ *R*_p.i.m._ = 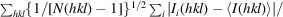 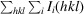 .

**Table 3:** Structure-solution and refinement statistics for the two complexes Values in parentheses are for the outer shell.

Complex	(+)-Anatoxin-a	(−)-Hosieine-A
Resolution range (Å)	59.77–2.50 (2.565–2.50)	45.03–2.60 (2.64–2.60)
Completeness (%)	99.5 (99.3)	100.0 (99.9)
No. of reflections		
Working set	113033 (8327)	102825 (7547)
Test set	6041 (449)	5457 (407)
Final *R*_cryst_ †	0.253 (0.335)	0.232 (0.291)
Final *R*_free_ ‡	0.289 (0.343)	0.257 (0.331)
Cruickshank DPI (Å)	0.409	0.421
No. of residues		
Protein	2049	2041
Ligand of interest	10	10
Water	957	434
Other ligands	Acetate, glycerol	Acetate
R.m.s. deviations		
Bond lengths (Å)	0.007	0.011
Angles (°)	1.093	1.538
Average *B* factors (Å^2^)		
Protein	29.8/28.6/27.5/26.9/28.4	32.1/31.4/30.6/27.4/28.5
	26.4/25.5/27.8/25.5/24.3	27.6/27.3/29.5/26.9/29.2
Ligand of interest	21.9/21.2/25.1/22.2/25.3	21.1/29.0/24.0/23.8/17.9
	13.9/20.6/18.6/20.2/16.7	22.5/18.1/19.2/17.1/21.0
Water	23.5	19.9
Ramachandran plot		
Most favored (%)	97.39	98.0
Allowed (%)	2.61	2.0
PDB code	6sh0	6sgv

† *R*_work_ = 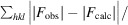 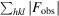 , where *F*_obs_ is the observed structure-factor amplitude and *F*_calc_ is the structure-factor amplitude calculated from the model.

‡ *R*_free_ was calculated with a subset of data that were excluded from refinement calculations (5%) using the same method as for *R*_merge_.

**Table 4:** Binding data for the interaction of *Ac*AChBP with (−)-nicotine, (+)-anatoxin-a, (−)-cytisine and (−)-hosieine-A Mean thermodynamic parameters are derived from ITC; the dissociation constant *K*_d1_ was determined from ITC and *K*_d2_ from fluorescence measurements. The standard error for each measurement is given (*n* = 3).

Ligand	*N* (sites)	Δ*G* (kcal mol^−1^)	Δ*H* (kcal mol^−1^)	−*T*Δ*S* (kcal mol^−1^)	*K*_d1_ (μ*M*)	*K*_d2_ (μ*M*)
(−)-Nicotine	0.4 ± 0.03	−7.7 ± 0.1	−17.8 ± 0.9	10.0 ± 0.9	2.30 ± 0.19	0.41 ± 0.01
(−)-Nicotine†	0.6	−8.3	−12.5	4.2	0.835	
(+)-Anatoxin-a	0.5 ± 0.02	−8.9 ± 0.1	−12.5 ± 0.2	3.6 ± 0.2	0.30 ± 0.03	0.15 ± 0.01
(−)-Cytisine†	0.6	−7.9	−13.3	5.4	1.6	
(−)-Cytisine‡	0.6 ± 0.01	−8.6 ± 0.3	−15.2 ± 1.2	6.6 ± 1.4	0.60 ± 0.03	
(−)-Hosieine-A	0.7 ± 0.03	−10.4 ± 0.2	−8.9 ± −0.1	−1.5 ± 0.3	0.025 ± 0.005	0.040 ± 0.001

† Data from Rucktooa *et al.* (2012 ▸) are presented for comparison.

‡ Data from Davis *et al.* (2020 ▸) are presented for comparison.
