# Supplementary material for: Delineating the activity of the potent nicotinic acetylcholine receptor agonists (+)-anatoxin-a and (−)-hosieine-A
Source: Acta Crystallogr F Struct Biol Commun. 2022 Aug 9;78(Pt 9):313–23. doi: 10.1107/S2053230X22007762 (PMC9435674; doi:10.1107/S2053230X22007762)
Supplement: Supplementary file 1 [file f-78-00313-sup1.pdf]

Acta Cryst F

STRUCTURAL BIOLOGY
COMMUNICATIONS

Volume 78 (2022)

Supporting information for article:

Delineating the activity of the potent nicotinic acetylcholine receptor agonists(+)-anatoxin-a and (−)-hosieine-A

Holly P. Parker, Alice Dawson, Mathew J. Jones, Rui Yan, Jie Ouyang, Ran Hong and William N. Hunter

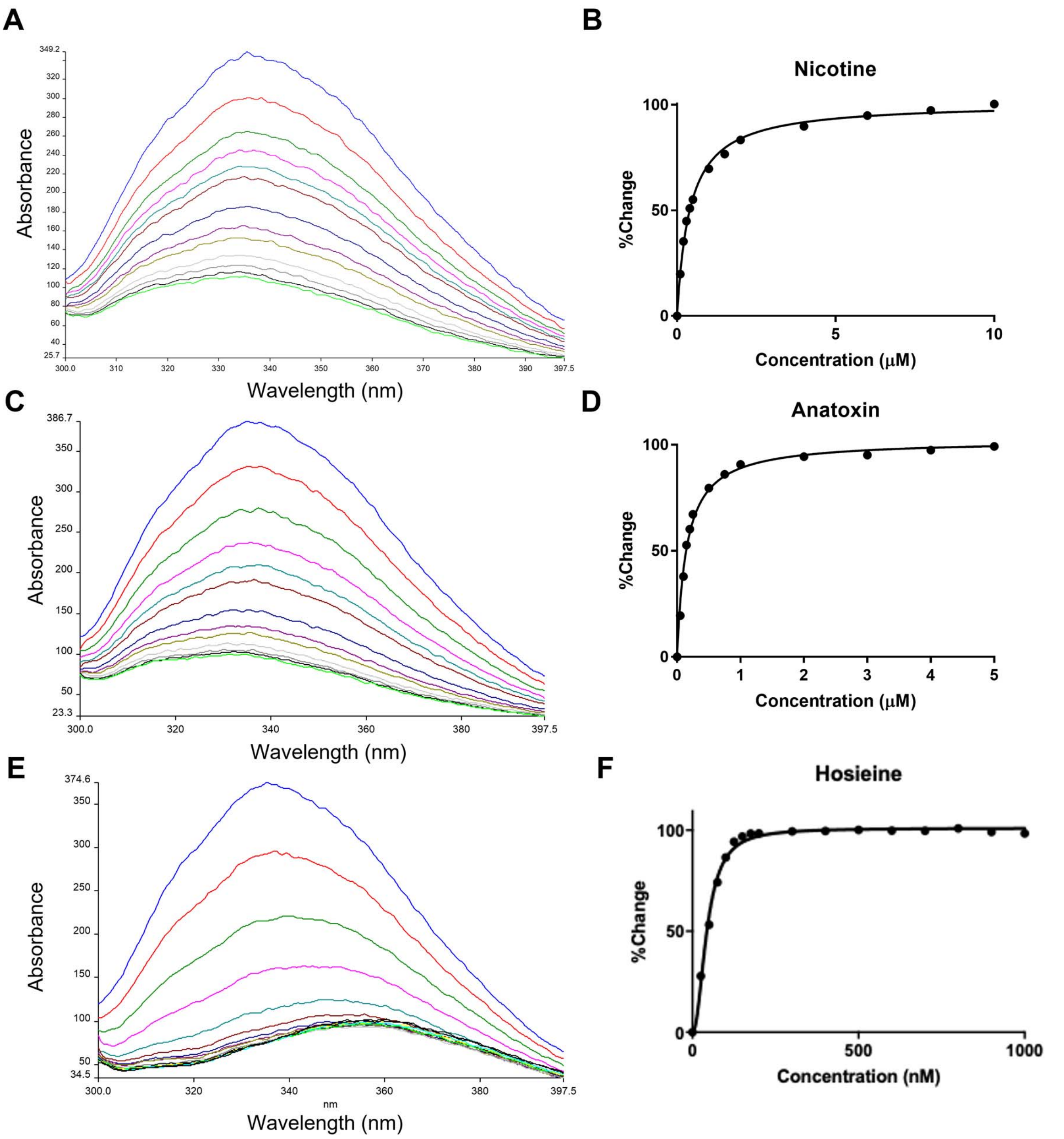

Figure S1 Ligand binding to *Ac*AChBP monitored by tryptophan fluorescence. Example traces obtained from titrations, starting with no ligand (blue) to maximum ligand concentration (green) and a plot of the mean percentage change versus concentration is shown for (-)-nicotine (A, B), (+)-anatoxin (C, D), and (-)-hosieine (E, F). Standard error bars are behind each data point (n=3).

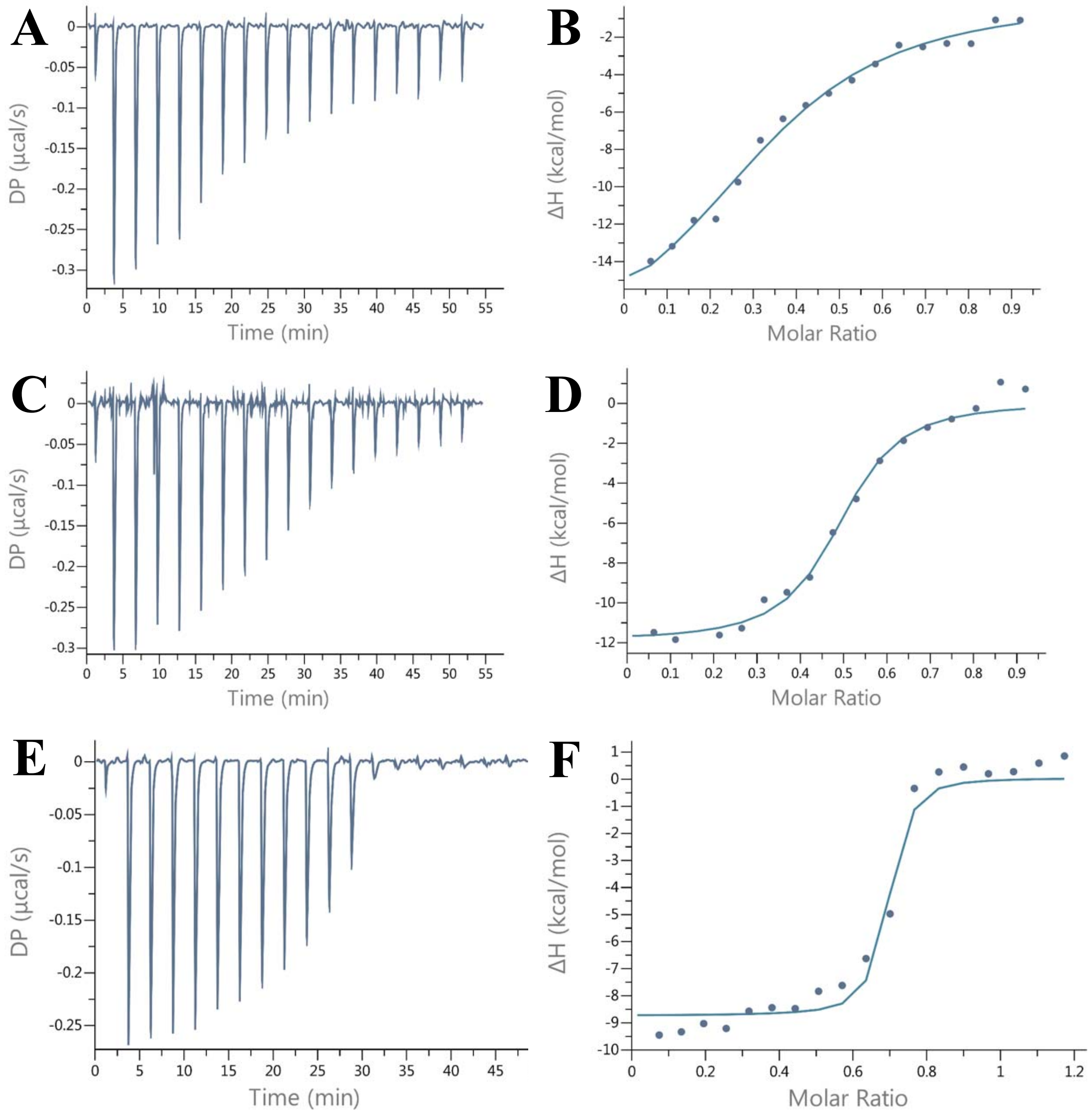

Figure S2 ITC data for *Ac*AChBP binding various ligands. Example isotherms and curves with the best fit are shown for (-)-nicotine (A, B), (+)-anatoxin-a (C, D) and (-)-hosieine-A (E, F), respectively. In the examples, a baseline deduction has been applied. All data have had the relevant controls (buffer-buffer, buffer-*Ac*AChBP, ligand-buffer) also deducted.

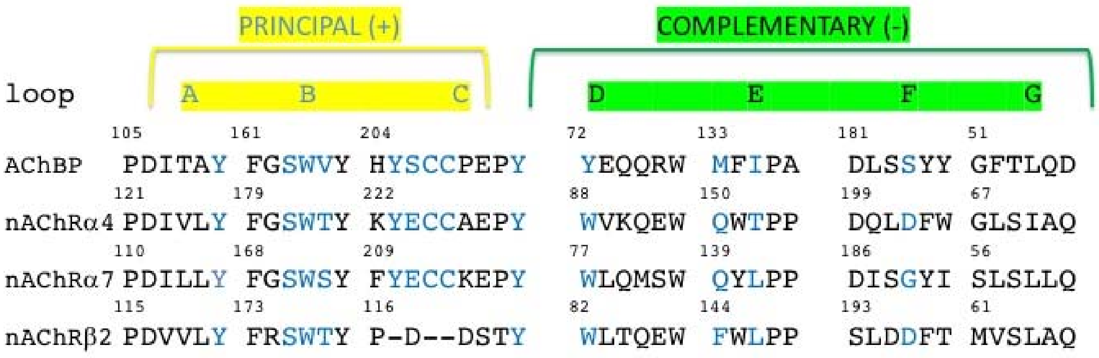

Figure S3 Alignment of selected sequence segments that form the orthosteric binding sites of *Ac*AChBP, human nAChR α4, α7 and β2. Segment loops are labelled and presented as principal (+) and complementary (-) sides. Residues colored light blue are discussed in text. Val125 and Thr127 of *Ac*AChBP are at the N-terminal section of loop E but are left out for the purpose of clarity.
